# Inhibition of Notch1-mediated inflammation by intermedin protects against abdominal aortic aneurysm via PI3K/Akt signaling pathway

**DOI:** 10.18632/aging.202436

**Published:** 2021-02-01

**Authors:** Xian-Qiang Ni, Ya-Rong Zhang, Li-Xin Jia, Wei-Wei Lu, Qing Zhu, Jin-Ling Ren, Yao Chen, Lin-Shuang Zhang, Xin Liu, Yan-Rong Yu, Mo-Zhi Jia, Zhong-Ping Ning, Jie Du, Chao-Shu Tang, Yong-Fen Qi

**Affiliations:** 1 Laboratory of Cardiovascular Bioactive Molecule, School of Basic Medical Sciences, Peking University, Beijing 100083, China; 2 Key Laboratory of Molecular Cardiovascular Science, Ministry of Education, Peking University Health Science Center, Beijing 100083, China; 3 Department of Pathogen Biology, School of Basic Medical Science, Peking University, Beijing 100083, China; 4 Key Laboratory of Remodeling-Related Cardiovascular Diseases, Beijing Institute of Heart, Lung, and Blood Vessel Diseases, Beijing An Zhen Hospital, Capital Medical University, Ministry of Education, Beijing 100029, China; 5 Shanghai University of Medicine and Health Sciences, Shanghai University of Medicine and Health Sciences Affiliated Zhoupu Hospital, Shanghai 201318, China

**Keywords:** abdominal aortic aneurysm, intermedin, Notch1, IMD transgenic and knockout mice, ADAM10

## Abstract

The Notch1-mediated inflammatory response participates in the development of abdominal aortic aneurysm (AAA). The vascular endogenous bioactive peptide intermedin (IMD) plays an important role in maintaining vascular homeostasis. However, whether IMD inhibits AAA by inhibiting Notch1-mediated inflammation is unclear. In this study, we found Notch intracellular domain (NICD) and hes1 expression were higher in AAA patients’ aortas than in healthy controls. In angiotensin II (AngII)-induced AAA mouse model, IMD treatment significantly reduced AAA incidence and maximal aortic diameter. IMD inhibited AngII-enlarged aortas and -degraded elastic lamina, reduced NICD, hes1 and inflammatory factors expression, decreased infiltration of CD68 positive macrophages and the NOD-like receptor family pyrin domain containing 3 protein level. IMD inhibited lipopolysaccharide-induced macrophage migration *in vitro* and regulated macrophage polarization. Moreover, IMD overexpression significantly reduced CaCl_2_-induced AAA incidence and down-regulated NICD and hes1 expression. However, IMD deficiency showed opposite results. Mechanically, IMD treatment significantly decreased cleavage enzyme-a disintegrin and metalloproteinase domain-containing protein 10 (ADAM10) level. Pre-incubation with IMD_17-47_ (IMD receptors blocking peptide) and the phosphatidylinositol 3-kinase/protein kinase b (PI3K/Akt) inhibitor LY294002 reversed ADAM10 level. In conclusion, exogenous and endogenous IMD could inhibit the development of AAA by inhibiting Notch1 signaling-mediated inflammation via reducing ADAM10 through IMD receptor and PI3K/Akt pathway.

## INTRODUCTION

Abdominal aortic aneurysm (AAA) is a fatal vascular disease defined as more than 50% arterial dilation of the abdominal aorta [1]. In recent years, the incidence of AAA has increased and it has become one of the top 10 causes of death among people older than 65 years. AAA is a result of the interaction by the environment, heredity and biochemistry and is characterized by chronic inflammation in the vascular wall [2]. Blood vessel wall damage caused by AAA could initiate the immune response, leading recruiting macrophages, T cells and mast cell infiltration to the vascular wall [3]. Then reactive oxygen species production, vascular smooth muscle cell (VSMC) apoptosis and matrix metalloproteinase (MMP) activation increases and leads to extracellular matrix degradation and remodeling, which is the main known pathogenesis factors of AAA [2, 4].

The general treatment of AAA includes classical open surgery and endovascular aneurysm repair. However, the survival rate and quality of life are not significantly improved by early surgical treatment [5–7]. There are still no effective clinical drugs to treat AAA [6] and exploring new pathogenesis factors to find novel therapeutic targets and drug treatment methods for AAA are crucial.

The Notch signal is highly expressed in invertebrates and vertebrates; it mainly consists of the Notch receptor and its ligand, delta-like canonical Notch ligand 4 (DLL4), which are both single transmembrane [8, 9]. When binding to its ligand, the Notch receptor is cut by a disintegrin and metalloproteinase (ADAM) protease and γ-secretase to release the Notch intracellular domain (NICD) with a nuclear positioning signal [10, 11]. The Notch1 signaling pathway plays an important role in regulating the development and transformation of various inflammatory cells such as macrophages and T lymphocytes, and it can modulate the immune response via nuclear factor κB (NF-κB) and phosphatidyl inositol 3-kinase/proteinkinase b (PI3K/Akt) pathways [12–14]. Notch1 signaling also plays a key role in diverse cardiovascular diseases such as valve calcification and left-sided congenital heart disease [8]. The inflammatory response mediated by Notch1 signaling pathway stabilizes the progression of small abdominal aortic aneurysm induced by angiotensin II (AngII) [14]. Furthermore, VSMC-specific Notch1 knockout could prevent AAA by regulating the contraction phenotype of VSMCs and maintaining extracellular matrix homeostasis [15]; therefore, Notch1 may have a pivotal role in AAA formation.

Recently, numerous studies, including our previous work, revealed that paracrine/autocrine factors play a vital role in AAA [16, 17, 27, 28]. For example, angiotensin-converting enzyme 2 [16] and epoxyeicosatrienoic acids [17] are both endogenous vasoactive factors that have benefit effect in suppressing AAA. A crucial vasoactive substance, intermedin (IMD), has been reported to exert important cardiovascular protection effects. IMD is a vasoactive peptide that belongs to the calcitonin gene-related peptide (CGRP) superfamily, discovered by Roh [18] and Takei [19] in 2004. The human IMD gene is located at the end of chromosome 22; it encodes a prepro-IMD consisting of 148 amino acid residues and generates 3 active fragments by proteolysis: IMD_1-47_, IMD_8-47_ and IMD_1-53_. IMD_1-53_ is likely the main active fragment of IMD [20]. Through the common receptor of CGRP and calcitonin receptor-like receptor/receptor activity-modifying protein receptor complexes (CRLR/RAMPs), IMD can increase intracellular cAMP content and participate in various biological effects such as myocardial contraction, vasodilation, regulation of cell proliferation, hypertrophy, migration and apoptosis [21–23]. IMD can also protect the endothelial barrier function and suppress local vascular inflammatory response [21]. In mammals, IMD attributes to protecting against cardiovascular disease such as atherosclerosis, myocardial ischemia-reperfusion injury, cardiac hypertrophy, vascular calcification and AAA [24–28]. Our previous work showed that IMD could restrain the formation of AAA by alleviating oxidative stress and endoplasmic reticulum stress [27, 28]. However, the mechanism of IMD inhibiting AAA has not been systematically studied.

In this study, we explored whether IMD can attenuate AAA by inhibiting Notch1 in AngII- and calcium chloride (CaCl_2_)-induced mouse models.

## RESULTS

### Notch1 signaling was activated in murine and human AAA

**Figure 1:**
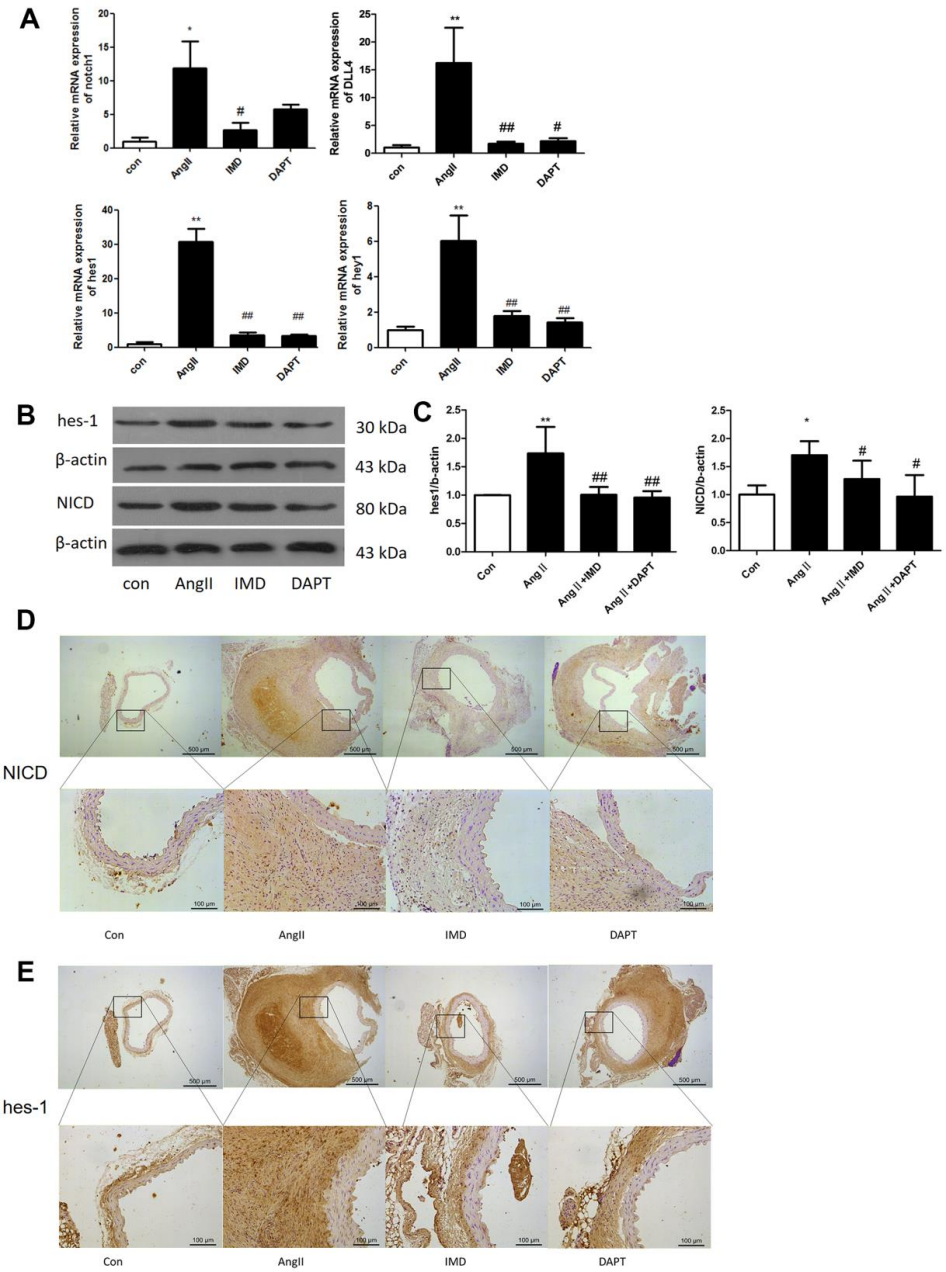
**Notch1 signaling was activated in murine AAA.** (**A**) Quantitative real-time PCR of Notch1, DLL4, hes1 and hey1 mRNA expression in aortas of mice with control (saline), AngII, AngII plus IMD and AngII plus DAPT. (**B**) Western blot analysis of hes1 and Notch1 signaling pathway marker NICD in aortas with Control, AngII, AngII+IMD and AngII+DAPT treatment. β-actin was a control for protein loading. Results are from 1 representative experiment of 3. (**C**) Quantification is shown as a ratio of β-actin expression. (**D**, **E**) Immunohistochemistry of the protein expression of NICD and hes1 in aortas of mice. Scale bar, 500 μm, 100 μm. Boxes and arrows show enlarged areas. Data are mean ± SD. *P<0.05, **P<0.01 vs. Control. ^#^*P*<0.05, ^##^*P*<0.01 vs. AngII.

**Figure 2:**
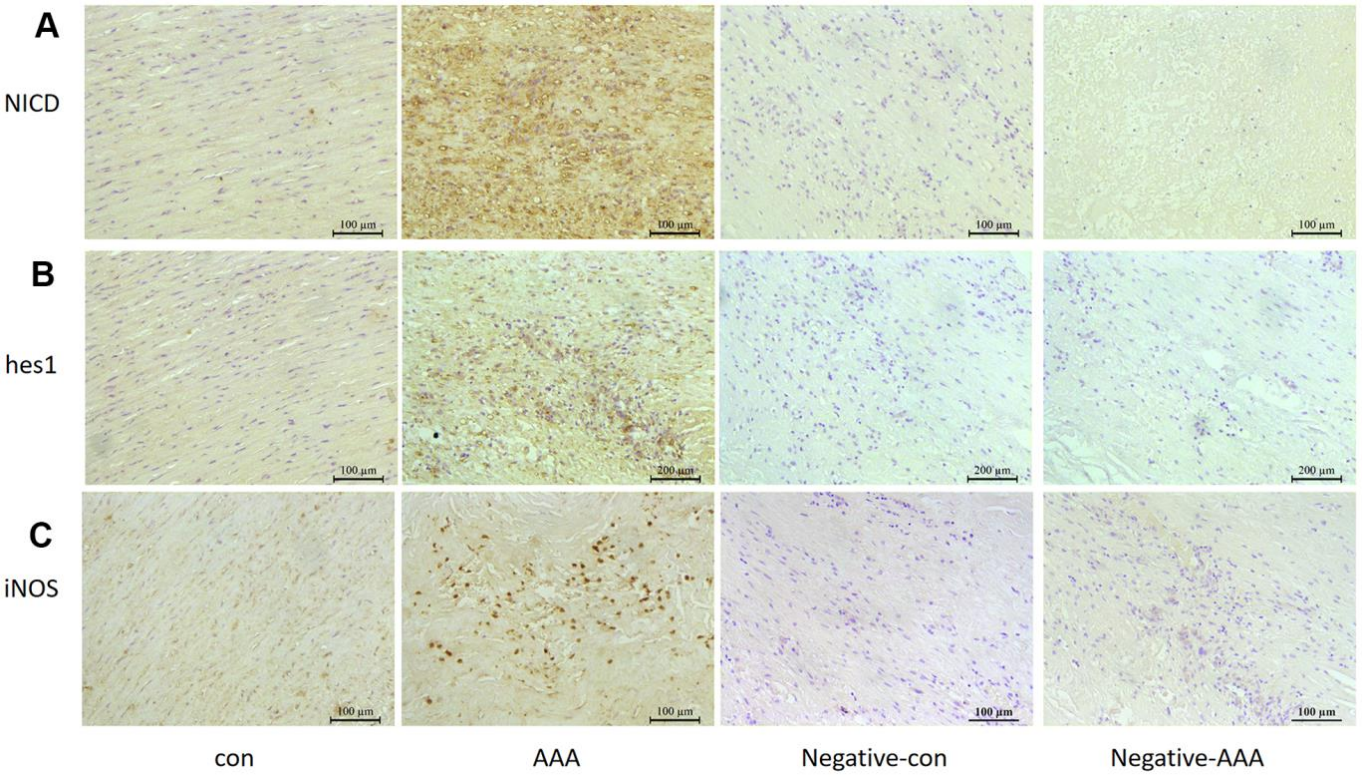
**Notch signaling and M1 polarization were activated in human aorta of AAA.** Control, Patients with non-abdominal aortic aneurysm; AAA, Patients with abdominal aortic aneurysm; Negative-con, Negative control group of patients with non-abdominal aortic aneurysm; Negative-AAA, Negative control group of patients with abdominal aortic aneurysm. Immunohistochemistry of (**A**, **B**) the protein expression of NICD and hes1 in patient aortas and (**C**) the protein expression of M1-type macrophage marker iNOS in patient aortas.

Notch1 signaling participates in local inflammation in the vessel wall, and inhibition of Notch1 signaling reduced AAA in mice by attenuating macrophage-mediated inflammation [14]. First, we detected Notch1 signaling markers in AngII-induced AAA mouse models. The mRNA levels of Notch1 and its ligand DLL4 were higher by 12.3- and 16.7- fold (both *P*<0.05) with than without AngII treatment. Meanwhile, Notch1 signaling target genes *hes1* and *hey1* were increased 30.5- and 5.92-fold (both *P*<0.05) with AngII treatment to induce AAA (Figure 1A). The protein levels of hes1 and the Notch1 active fragment NICD were increased by 76.4% and 67.1% (both *P*<0.05) with AAA (Figure 1B, 1C). Immunohistochemistry results were consistent with western blot findings, and hes1 and NICD protein were mainly located in vascular adventitia (Figure 1D, 1E). Furthermore, levels of Notch1 signaling markers NICD, hes1 and inducible nitric oxide synthase (iNOS) were obviously higher in human AAA aortas than in normal aortas (Figure 2A–2C).

### IMD alleviated AngII-induced AAA by blocking Notch1 signaling

**Figure 3:**
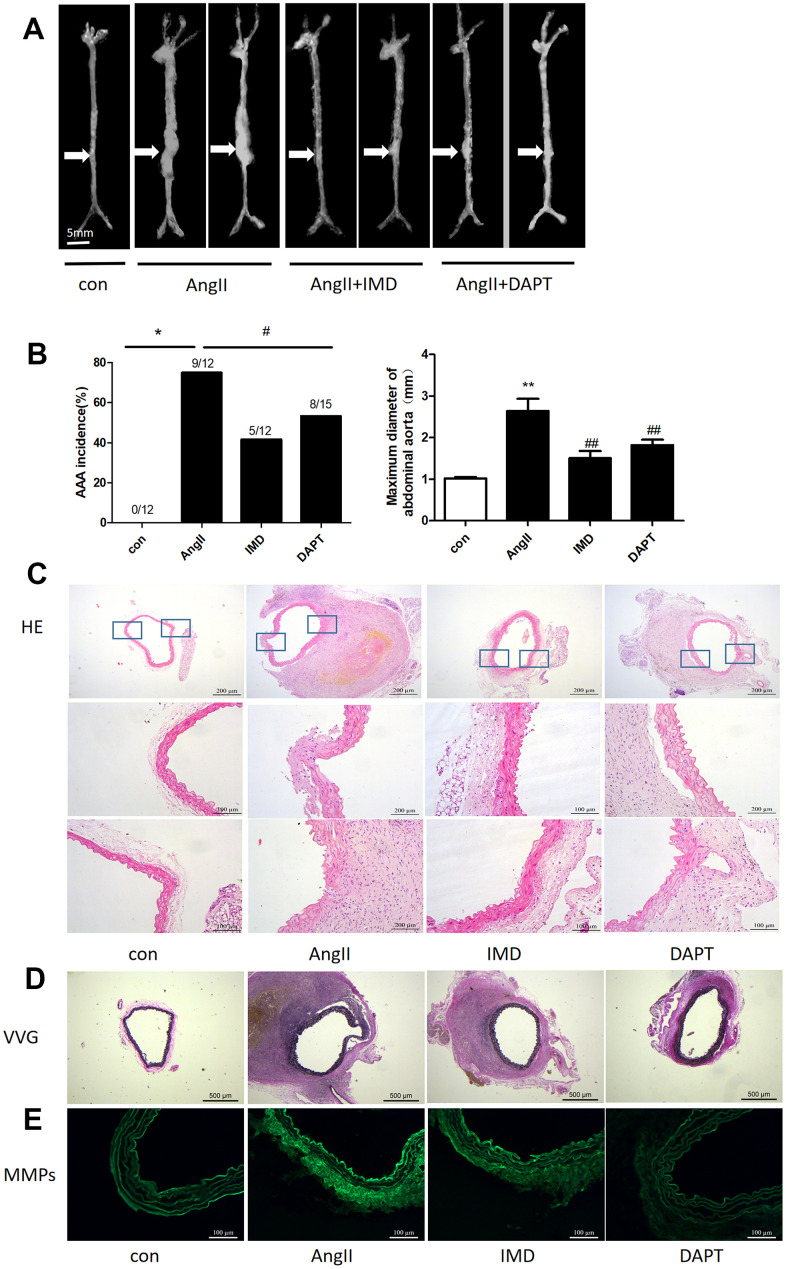
**IMD alleviated AngII-induced AAA in mice by blocking Notch1 signaling**. (**A**) Representative photographs of macroscopic features of AAA in male ApoE-/- mice. Control, saline infusion; AngII, AngII (1000 ng/kg/min) infusion; AngII+IMD (300 ng/kg/min) infusion; AngII+DAPT (10 mg/kg) gavage for 4 weeks. The arrows indicate typical AAA in ApoE-/- mice. Scale bars, 5 mm. (**B**) Incidence of AngII-induced AAA in animals and maximal abdominal aortic diameter of survivor mice at the end of 28 days. (**C**) Representative HE staining of aortas. Scale bar, 200 μm, 100 μm. (**D**) Representative VVG staining. Scale bar, 500 μm. (**E**) Representative in situ zymography of MMP activity in suprarenal aortas from ApoE-/- mice. Scale bar, 100 μm. Data are mean ± SD. *P<0.05, **P<0.01 vs. Control. #P<0.05, ##P<0.01 vs. AngII.

Then, we tested the effects of IMD using AngII-induced AAA model. Negative control animals treated with saline did not show AAA features. The morbidity with AngII treatment was 75%, which was consistent with previous studies [28]. IMD treatment significantly reduced the occurrence of AAA to 46.2% (*P*<0.05) and N-[N-(3,5-difluorophenacetyl)-Lalany]-S-phenylglycine t-butyl ester (DAPT) (Notch1 pathway inhibitor) had a similar effect as IMD, with 53.3% AAA morbidity (*P*<0.05) (Figure 3A, 3B). The maximum abdominal aorta diameter of AngII-treated mice was markedly dilated as compared with saline treatment and was reversed with IMD and DAPT treatment (Figure 3B). AAA aortas showed intramural thrombus formation, severe aortic dilatation (Figure 3C), elastin degradation (Figure 3D) and increased MMP activity (Figure 3E), which were all alleviated with IMD infusion. DAPT also had a similar inhibitory effect as IMD.

Next we explored whether IMD protected against AAA formation by inhibiting Notch1 signaling activation. Notch1 signaling markers were upregulated during AAA induction (Figure 1). After IMD treatment, the mRNA expression of Notch1, DLL4, *hes1* and *hey1* were decreased in abdominal aortas 81.3%, 86.8%, 91.2% and 70.1%, respectively (all *P*<0.05) (Figure 1A), as compared with AngII treatment. Protein levels of hes1 and NICD were reduced 45.6% and 29.6% (both *P*<0.05) as compared with AngII treatment (Figure 1B, 1C). Immunohistochemistry showed similar results (Figure 1D, 1E). DAPT had similar effects as IMD.

### IMD inhibited macrophage-mediated inflammation by Notch1 signaling

**Figure 4:**
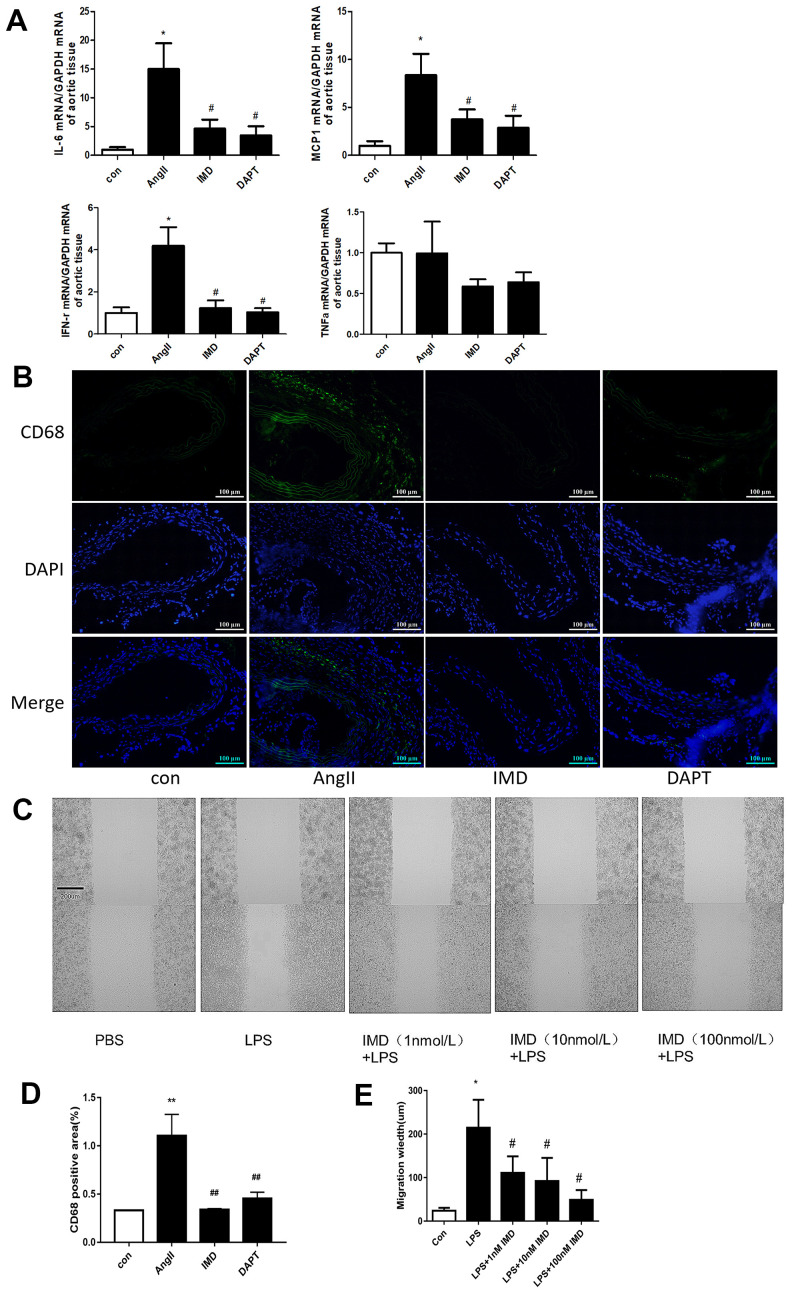
**IMD inhibited macrophage-mediated inflammation via Notch1 signaling.** (**A**) Quantitative real-time PCR of mRNA levels of IL-6, MCP-1, IFN-γ and TNF-α in aortas of Control, AngII, AngII+IMD and AngII+DAPT mice. (**B**) Representative immunofluorescence analysis of the protein expression of CD68 (green) and DAPI (blue) in aortas of mice. Scale bar, 100 μm. (**C**) Scratch-wound test to detect the transfer ability of mouse peritoneal macrophages in vitro. PBS, control group; LPS, stimulated group (100 ng/mL, 12 h). IMD was pretreated with a concentration gradient (1, 10, 100 nmol/L, 1 h). Cell migration distance was observed at 12 h after scratch wounding. Scale bar, 200 μm. n=3. (**D**) Quantification of (**B**). n=3, Data are mean ± SD. **P<0.01 vs. Control. ^##^*P*<0.01 vs. AngII. (**E**) Quantification of scratch-wound test. n=3, Data are mean ± SD. *P<0.05 vs. Control. ^#^*P*<0.05 vs. LPS.

Notch1 signaling-mediated macrophage inflammation plays a critical role in AAA pathogenesis [14]. Since activated Notch1 signaling markers were mainly located in vascular adventitia (Figure 1D, 1E), so we supposed that IMD could inhibit AAA by alleviating macrophage-mediated inflammation. First, we detected the expression of representative inflammatory factors in mouse aortas. The mRNA levels of IL-6, monocyte chemotactic protein-1 (MCP-1) and interferon (IFN-γ) were significantly elevated after AngII infusion; IMD and DAPT reversed their expression (Figure 4A). However, the expression of tumor necrosis factor-α (TNF-α) was not changed. It was reported that IL-6 and MCP-1 were the strongest inflammatory factors involved in macrophage migration [3], so we examined the infiltration of macrophages in the vascular wall. Immunofluorescence assay showed that the expression of CD68, a typical macrophage marker, was higher in the vascular wall with AngII than control treatment; IMD and DAPT remarkably reduced the CD68 fluorescence intensity (Figure 4B, 4D). *In vitro*, using wound-healing assay, lipopolysaccharide (LPS) treatment significantly increased the migration distance of peritoneal macrophages as compared with control treatment; IMD pretreatment could dose-dependently inhibit the migration of macrophages (Figure 4C, 4E).

### IMD inhibited NOD-like receptor family pyrin domain containing 3 (NLRP3) inflammasome activation and M1 polarization via Notch1 signaling

**Figure 5:**
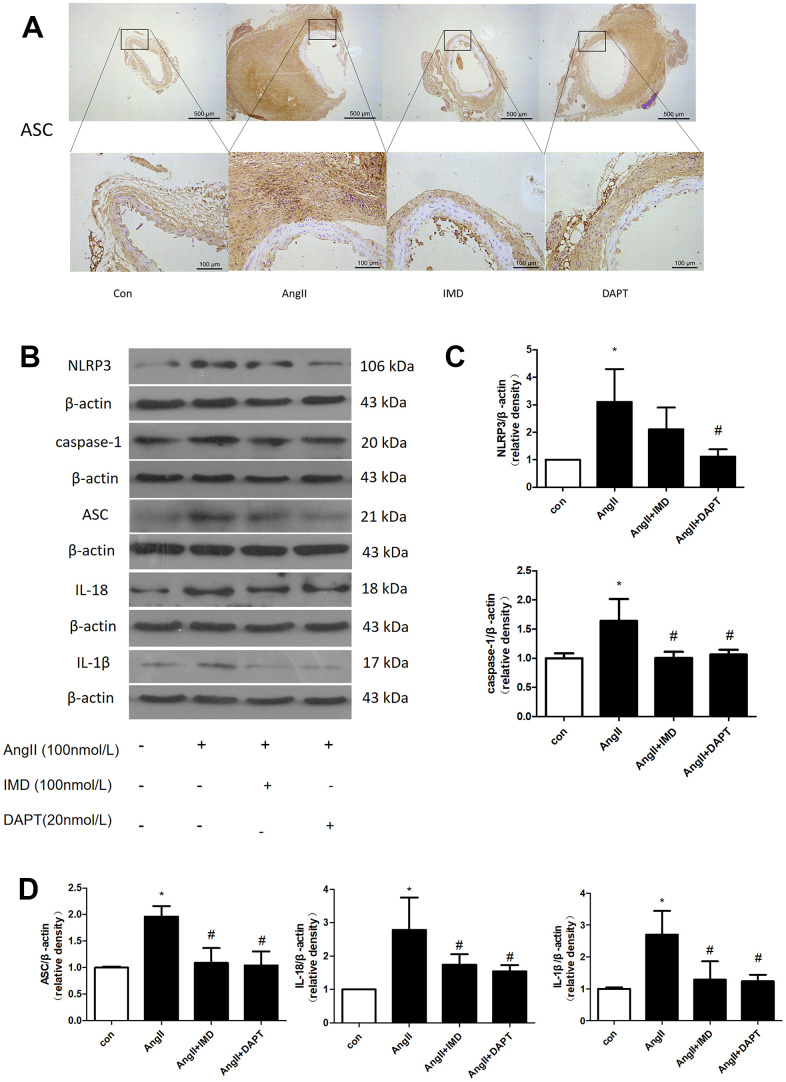
**NLRP3 inflammasome activation was inhibited by IMD via Notch1 signaling.** (**A**) Immunohistochemistry analysis of the protein expression of ASC in aortas of Control, AngII, AngII+IMD and AngII+DAPT mice. Control, saline infusion; AngII, AngII (1000 ng/kg/min) infusion; AngII+IMD (300 ng/kg/min) infusion; AngII+DAPT (10 mg/kg) gavage for 4 weeks. Scale bar, 500 μm, 100 μm. Boxes and arrows show enlarged areas. (**B**) Western blot analysis of protein levels of NLRP3, ASC, caspase-1, IL-1β, IL-18 in Raw264.7 macrophage cell line. The cell line was exposed to PBS, AngII (100 nm, 1 h) and DAPT (20 nm, 1 h). (**C**, **D**) Quantification of (**B**). n=3, Data are mean ± SD. *P<0.05 vs. Control. ^#^*P*<0.05 vs. AngII.

NLRP3 inflammasome activation leads to production of mature IL-1β and IL-18, then recruitment of a large number of inflammatory cells to the vascular wall, which plays an important role in the initiation of inflammatory responses in the early stages of various diseases [29]. To investigate whether the protective effects of IMD against AAA formation are also mediated by NLRP3 inflammasome activation, we examined the apoptosis-associated speck-like protein containing CARD (ASC) expression. Immunohistochemical results showed that the expression of ASC in abdominal aortic tissues was increased in the AngII aneurysm group and reduced with IMD and DAPT treatment (Figure 5A). Next, we further explored the effect of IMD on NLRP3 inflammasome activation in a macrophage line Raw264.7. The protein level of NLRP3, ASC, caspase-1, IL-18, and IL-1β was upregulated during AAA formation, and IMD and DAPT inhibited NLRP3 inflammasome activation (Figure 5B–5D).

**Figure 6:**
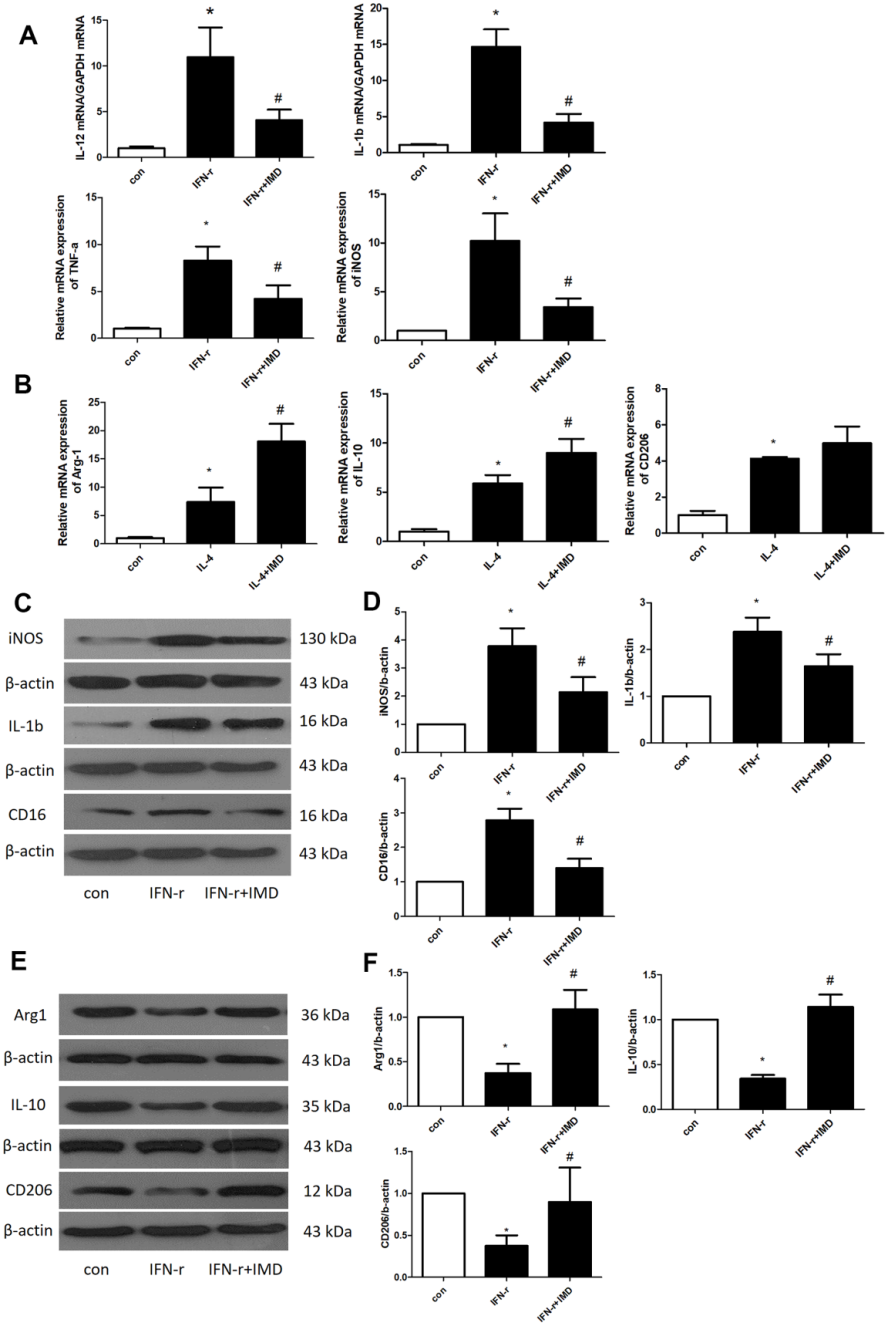
**IMD inhibited macrophage M1 polarization.** Control, saline treatment group; LPS+ IFN-γ, M1-type macrophage induction group; IFN-γ+IMD, M1-type macrophages pretreated with IMD; IL-4, M2-type macrophage induction group; IL-4+IMD, M2-type macrophages pretreated with IMD. Quantitative real-time PCR analysis of mRNA levels of (**A**) M1-type macrophage marker IL-12, IL-1β, TNF-α and iNOS in BMDMs and (**B**) M2-type macrophage marker Arg1, IL-10 and CD206 in BMDMs. Western blot analysis of (**C**, **D**) M1-type macrophage markers IL-12, IL-1β and CD16 and (**E**, **F**) M2-type macrophage markers Arg1, IL-10 and CD206 in BMDMs. β-actin was a control for protein loading. n=3, Data are mean ± SD. *P<0.05 vs. Control. ^#^*P*<0.05 vs. IFN-γ.

Under different microenvironments, macrophages can be polarized to assume different phenotypes and show diverse functions. Generally, M1-type macrophages are considered to have pro-inflammatory phenotypes and M2-type macrophages anti-inflammatory phenotypes [30]. M1 and M2 macrophages polarization contribute to AAA formation [2, 3]. Therefore, we detected the effect of IMD on macrophage polarization in bone-marrow-derived macrophages (BMDMs). IFN-γ and interleukin-4 (IL-4) were used to induce BMDM M1 and M2 polarization, respectively. The mRNA expression of the M1 polarization markers IL-12, IL-1β, TNF-α and iNOS was greatly upregulated after IFN-γ treatment; IMD inhibited M1 polarization marker expression (Figure 6A). Similarly, IL-4 promoted the expression of the M2 polarization markers arginase 1 (Arg1), IL-10 and CD206, and IMD further facilitated their expression (Figure 6B). We further explored the protein level of M1 and M2 markers. IFN-γ increased the M1 marker protein levels of iNOS, IL-1β and CD16, and IMD suppressed the protein levels (Figure 6C, 6D). In contrast, IFN-γ suppressed the M2 protein levels of Arg1, IL-10 and CD206, and IMD restored the levels (Figure 6E, 6F). In mice AAA tissue, the M1 macrophage polarization marker iNOS was increased in Ang II group compared with control, IMD and DAPT could decrease the iNOS expression; M2 macrophage polarization marker Arg1 was activated in Ang II group, which increased more in IMD and DAPT group (Supplementary Figure 2A, 2B).

### IMD restrained Notch1 signaling activation by suppressing the expression of ADAM10

**Figure 7:**
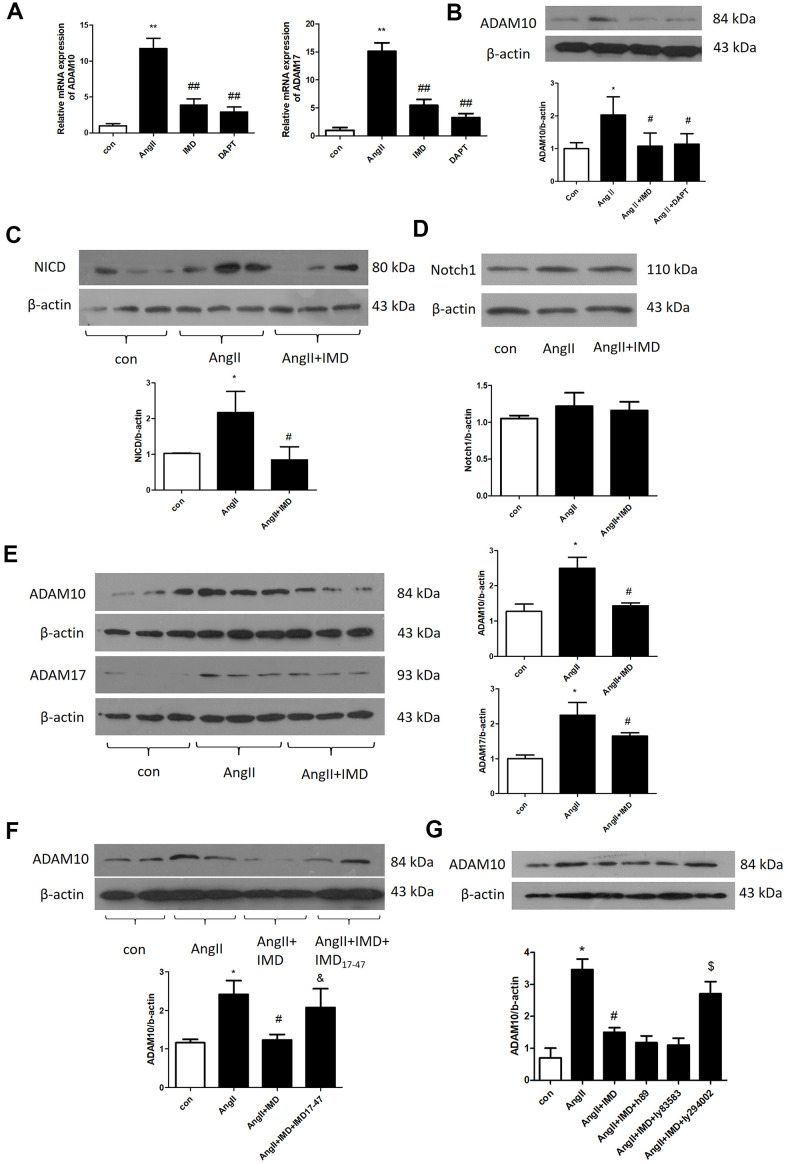
**IMD inhibited Notch1 signaling activation by ADAM10.** (**A**) Quantitative real-time PCR analysis of mRNA levels of ADAM10 and ADAM17 in aortas of Control, AngII, AngII+IMD and AngII+DAPT in mice. (**B**) Western blot analysis of protein level of ADAM10 in aortas of Control, AngII, AngII+IMD and AngII+DAPT mice. Western blot analysis of protein levels of (**C**, **D**) NICD, the intracellular fragment of Notch1 and the full length Notch1 receptor and (**E**) ADAM10 and ADAM17 in AngII-induced macrophage cell line Raw264.7. n=3, Data are mean ± SD. *P<0.05, **P<0.01 vs. Control. ^#^*P*<0.05, ^##^*P*<0.01 vs. AngII. (**F**) Western blot analysis of protein level of ADAM10 in AngII-induced macrophage cell line Raw264.7. Except for the first 3 groups, IMD receptor blocker IMD_17-47_ group was added. n=3, Data are mean ± SD. *P<0.05 vs. Control. ^#^*P*<0.05 vs. AngII. ^&^ *P*<0.05 vs. AngII+IMD. (**G**) Western blot analysis of protein level of ADAM10 in AngII-induced macrophage cell line Raw264.7. Except for the first 3 groups, cAMP/PKA inhibitor H89, cGMP/PKG inhibitor Ly83583 and PI3K/Akt inhibitor LY294002 were added. n=3, Data are mean ± SD. *P<0.05 vs. Control. ^#^*P*<0.05 vs. AngII. ^$^ *P*<0.05 vs. AngII+IMD.

Next we explored the molecular mechanism of IMD inhibiting Notch1 signaling activation. ADAM10 and ADAM17 are key proteases during Notch1 signaling pathway activation [11]. In AngII-induced mice abdominal aortas, the mRNA expression of ADAM10 and ADAM17 was increased by 11.9- and 15.1-fold (both *P*<0.05). IMD decreased ADAM10 and ADAM17 mRNA expression by 68.7% and 70.1% (both *P*<0.05) (Figure 7A). Results for the protein level of ADAM10 were consistent with mRNA findings (Figure 7B).

We further explored the mechanism in a macrophage lineage Raw264.7. First, we detected the protein levels of NICD and full-length Notch1 receptors in Raw264.7 macrophages by western blot analysis. AngII treatment increased NICD level by 2.19-fold (*P*<0.05), which was reversed by IMD pre-infusion; Notch1 receptor level was similar in 3 groups (Figure 7C, 7D). Also, ADAM10 and ADAM17 protein levels were elevated by AngII and reversed by IMD in macrophages (Figure 7E). The level of Akt phosphorylation showed the opposite trend as NICD, ADAM10 and ADAM17 (Supplementary Figure 1A). Furthermore, IMD17-47, an IMD receptor blocker, intervened in the effect of IMD on ADAM10 expression (Figure 7F). The PI3K/Akt pathway inhibitor LY294002 also blocked the effect of IMD on ADAM10 expression, but cyclic adenosine monophosphate/proteinkinase A (cAMP/PKA) and cGMP/PKG inhibitors had no effect on ADAM10 expression (Figure 7G).

### Endogenous IMD alleviated CaCl_2_-induced AAA formation

**Figure 8:**
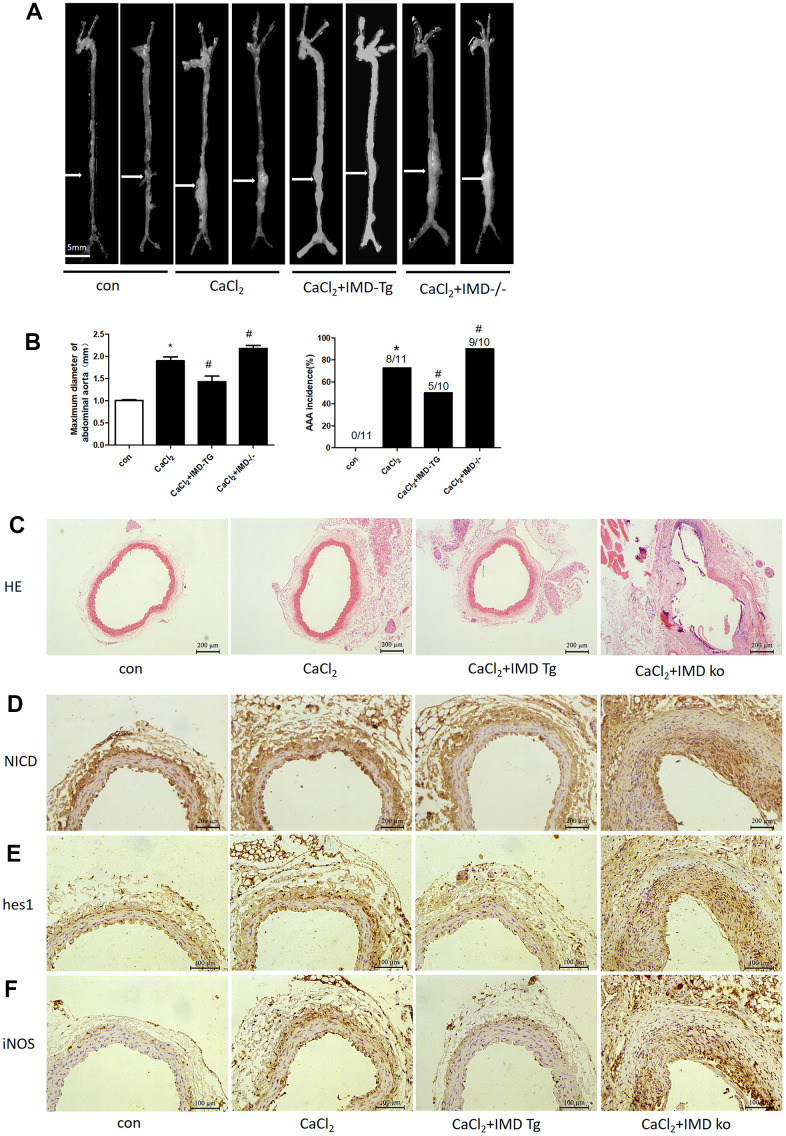
**Endogenous IMD alleviated CaCl_2_-induced AAA and Notch1 signaling and M1 polarization.** Control, wild type C57 mice with saline incubation; CaCl_2_, wild-type C57 mice with CaCl_2_ incubation; CaCl_2_+IMD-Tg, IMD transgenic mice with CaCl_2_ incubation to induce AAA; CaCl_2_+IMD ko, IMD-knockout mice with CaCl_2_ incubation to induce AAA. (**A**) Representative photographs of macroscopic features of aneurysms in male C57 mice at the end of 28 days. Scale bar, 5 mm. (**B**) Incidence of CaCl_2_-induced AAA in animals and maximal abdominal aortic diameter of surviving mice at the end of 28 days. The vascular diameter > 50% of the average diameter of the control group was considered AAA. *P<0.05 vs. Control. ^#^*P*<0.05 vs. CaCl_2_. (**C**) Representative HE staining. Scale bar, 200 μm. (**D**–**F**) Immunohistochemistry of the protein expression of NICD, hes1 and iNOS in aortas of mice.

Our previous studies reported that exogenous IMD protected against AAA formation by inhibiting oxidative stress and endoplasmic reticulum stress [27, 28]. To further explore the role of endogenous IMD in AAA, we created IMD transgenic and knockout mice and induced aneurysm by incubation of CaCl_2_ in the abdominal aorta. With CaCl_2_ incubation, AAA was induced in 73% of mice in a C57 background (Figure 8A, 8B), the maximum abdominal aorta diameter was increased and the protein levels of NICD, hes1 and M1 macrophage marker iNOS were upregulated (Figure 8A, 8B, 8D, 8E, 8F). Moreover, 90% of IMD-knockout mice (*P*<0.05) developed AAA and showed more increased maximum abdominal aorta diameter (Figure 8A, 8B). However, AAA was induced in only 50% of IMD transgenic mice (*P*<0.05) and the maximum abdominal aorta diameter was decreased (Figure 8A, 8B). HE staining showed that CaCl_2_ induction caused dilated abdominal aorta, vascular wall thickening and damaged vascular elastic plates, which was alleviated by IMD overexpression and aggravated by IMD deficiency (Figure 8C). Moreover, Notch1 pathway markers were upregulated in aortas of IMD-knockout mice and were downregulated in IMD transgenic mice (Figure 8D–8F).

## DISCUSSION

In the present study, the Notch1 signaling pathway was significantly activated during the pathogenesis of AAA. As an important vasoactive factor, exogenous and endogenous IMD could significantly decrease the occurrence and development of AAA by suppressing the Notch1 signaling pathway. IMD inhibited the expression of the protease ADAM10 through the PI3K/Akt signaling pathway via a CRLR/RAMP receptor and thereby inhibited activation of the Notch1 signaling pathway. Suppression of Notch1 signaling pathway by IMD further prevented NLRP3 activation and M1 polarization of macrophages, promoted M2 macrophage polarization, thus reducing the inflammatory response in local aortas to prevent AAA formation. Thus, IMD might be a vital target for treating AAA in clinical practice.

First, we investigated the regulatory role of exogenous IMD in an AngII-induced AAA model in ApoE^-/-^ mouse background. AngII can affect endothelial cell function, elevate blood pressure, and promote macrophage activation and pro-angiogenesis to accelerate AAA formation [28, 31]. As a classical model of AAA, the AngII-induced mice model is widely used in studying the pathogenesis of AAA, simulating most of the pathological features in human AAA, such as atherosclerosis plaque and thrombosis, infiltration of inflammatory cells in the local vasculature, and severe destruction of vascular elastin [32, 33]. Our results were consistent with those in the literature. Thus, IMD has a protective effect in the development of AAA.

Then we explored how IMD inhibited AAA. As shown in previous studies, Notch signal plays an important role in the pathogenesis of various diseases, such as tumor and cardiovascular diseases [34]. Notch1 affects vascular calcification and participates in the phenotypic changes of vascular smooth muscle cells via multiple signaling pathways [35, 36]. Here, we found that the levels of the Notch1 pathway marker NICD and hes1 were increased in the vascular tissues of patients with AAA, suggesting the Notch1 signaling pathway activation. We also found increased expression of NICD and hes1 in aortas of AngII-induced mice; IMD infusion could significantly inhibit this protein activation. We also used DAPT, a Notch1 inhibitor, which had similar inhibitory effect as IMD. So IMD might prevent AAA via the Notch1 signaling pathway.

AAA is a chronic vascular disease characterized by a local invasive inflammatory response. Inflammatory cells infiltrate the vessel wall and release a large number of inflammatory factors and produce a large amount of reactive oxygen species, which is a critical process in the pathogenesis of aneurysm [37, 38]. Inflammatory cells, especially T lymphocytes and macrophages, are primarily located in the media and adventitia of the aorta [2] and produce a large amount of MMPs, inflammatory cytokines and chemokines, such as IL-1β, IL-6 and MCP-1, affecting extracellular matrix homeostasis and promoting the apoptosis of smooth muscle cells [39, 40]. We found that IMD could reverse the increased levels of inflammatory factors. Immunofluorescence assay showed increased expression of the macrophage marker CD68 in the vascular wall of mice with AAA and IMD reversed the CD68 level, which might be related to the impaired migration capacity of macrophages after IMD infusion, as testified by the scratch-wound assay. Therefore, the anti-inflammatory effect of IMD might be mediated by inhibiting Notch1.

NLRP3 is the most studied inflammasome and has extensive functions. Activation of NLRP3 needs two signals: Toll-like receptors as the first signal activate NF-κB [41], which increases the precursor expression of NLRP3, IL-1β and IL-18. When receiving the second signal, NLRP3 proceeds with oligomerization and recruits molecular ASC. ASC assembles filamentous structure recruitment and enables pro-caspase-1 to shear and activate, then produces the mature IL-1β and IL-18 from their precursors [29, 42]. Mitochondrial oxidative stress in macrophages can activate NLPR3, which plays a role in initiating the inflammatory response in early AAA formation [7]. In this study, AngII promoted NLRP3 activation both in AAA mouse model and cultured macrophages; both IMD and DAPT reversed NLRP3 expression, so IMD may inhibit the activation of NLRP3 in macrophages by blocking the Notch1 signaling pathway, thereby preventing the initiation of the early inflammatory response of AAA.

We also investigated the effect of IMD on macrophage polarization in mouse BMDMs. IMD pre-treatment significantly inhibited macrophage M1 polarization and promoted macrophage M2 polarization. Macrophages have diverse functional characteristics in different microenvironments, showing obvious heterogeneity. M1 macrophages, as the pro-inflammatory phenotype, have a strong promoting effect on inflammatory diseases such as AAA, whereas M2 macrophages can alleviate the local inflammatory response of vessels and have a certain protective effect on AAA [43–45]. IMD also inhibited AAA by regulating macrophage polarization in mice aortas. Overall, IMD may inhibit the formation of AAA by inhibiting M1-type macrophage polarization and promoting M2-type macrophage polarization.

We found that IMD could suppress AAA formation via the Notch1 signaling pathway and therefore restrain NLRP3 activation and macrophage M1 polarization. However, how IMD inhibited Notch1 needs elucidation. The activation of Notch1 is regulated by multiple signaling pathways. The binding of the ligand to the receptor leads to two protein cleavages of the receptor, which is the main mechanism of Notch signaling pathway activation. The first cleavage is mediated by members of the ADAM metalloproteinase family (mainly ADAM10 and ADAM17) and the second is completed by a protein-secreting enzyme complex. Proteases ADAM10 and ADAM17 have key regulatory effects on the activation of the Notch1 pathway [11]. In human patients with non-small cell lung cancer, ADAM10 overexpression activated the Notch1 signaling pathway, leading to increased cell migration and invasion [46]. ADAM17 could regulate the chemical sensitivity of colorectal cancer stem cells by activating the Notch1 signaling pathway [47]. Therefore, we hypothesized that regulation of the Notch1 signaling pathway by IMD was achieved via the proteases ADAM10 and ADAM17. The expression of ADAM10 and ADAM17 was significantly higher in the abdominal aortas of mice with AAA than controls and was significantly reduced after IMD treatment. In Raw264.7 macrophages, IMD significantly inhibited the protein level of NICD but had no significant effect on the full-length Notch1 receptor. These results indicate that IMD blocks the activation of the Notch1 signaling pathway by inhibiting the expression of the protease ADAM10.

We further explored the mechanism of IMD regulating the expression of ADAM10. IMD exerts its biological effects via nonselective interaction with the CRLR/RAMP receptor complex, and the ligand-binding selectivity of CRLR is regulated by RAMP1, 2, and 3 [20]. We found that IMD_14-47_, a CRLR/RAMP receptor blocker, and PI3K/Akt inhibitor LY294002 blocked the regulation of IMD on ADAM10 expression. Therefore, IMD could inhibit ADAM10 protein expression via its CRLR/RAMP receptor and the PI3K/Akt signaling pathway, thereby inhibiting the activation of Notch1.

In the present work we revealed the protective role of exogenous IMD in an AAA mouse model. To further explore the role of endogenous IMD in AAA, we constructed IMD transgenic and knockout mice in a C57/BL background. We used an AAA mouse model induced by CaCl_2_, characterized by a severe inflammatory response in local vessels [29]. As compared with wild-type mice, IMD transgenic mice showed significantly reduced incidence of AAA and average abdominal aorta maximum diameter, and IMD-knockout mice showed the reverse results. With CaCl_2_ incubation, the abdominal aorta was dilated, the vascular wall was thickened and vascular elastic plates were damaged; all were alleviated in IMD transgenic mice and aggravated in IMD-knockout mice. These results indicate that endogenous IMD plays a key role in inhibiting AAA and maintaining the homeostasis of vascular structure and function. Our previous study reported that IMD transgenic mice showed alleviated the neointima formation in a carotid-artery-ligation model [22]. In this study, we first confirmed the protective effect of endogenous IMD in AAA formation. We also detected the Notch1 pathway and M1 macrophage markers in IMD transgenic and knockout mice with CaCl_2_ incubation. These proteins were higher in aortas with IMD deficiency and downregulated with IMD overexpression, which suggests that endogenous IMD suppressed Notch1 signaling pathway activation and M1-type macrophage polarization in an AAA model induced by calcium chloride.

Two different AAA models were used in this study, Ang II induction for 4 weeks in ApoE^-/-^ background and CaCl_2_ incubation in C57 background mice. Ang II induced model is a classic AAA model and it has been widely used to study the pathogenesis of AAA and can simulate most of the pathological features of human AAA, such as atherosclerotic plaques and thrombosis, local vascular inflammatory cell infiltration and severe elastin damage [32, 33]. However, the formation of CaCl_2_ induced AAA is mainly dependent on the destruction of elastic fibers by calcium deposition in the medium and the activation of inflammatory response, which is mainly manifested as calcification, vascular smooth muscle cell apoptosis, infiltration of inflammatory cells in the adventitia and media and more serious degradation of elastins [48]. Both models showed significant inflammatory cell infiltration and elastin degradation. In this study, although the models were inconsistent, they both proved that the inflammation mediated by Notch1 signaling pathway was involved in the occurrence and development of AAA and IMD could protect AAA by inhibiting Notch1 mediated inflammation.

Numerous studies in animal models have shown significant therapeutic potential targets for treating AAA; however, there is still no effective target for clinical application [6]. So it is essential to find new targets. In our previous and present work, IMD has shown some benefits for inhibiting AAA. First, as an endogenous bioactive peptide, IMD is widely distributed in the cardiovascular system. Also, IMD could suppress early inflammatory response via the Notch1 signaling pathway to prevent AAA to achieve the purpose of early treatment. Nevertheless, the mechanism by which IMD inhibits Notch1 needs further exploration.

Taken together, our results provide evidence that exogenous and endogenous IMD can reduce the occurrence of AAA by inhibiting Notch1 signaling pathway-mediated inflammation. IMD binds to its receptor non-selectively to suppress ADAM10 protein level via the PI3K/Akt signaling pathway, thereby restraining the activation of Notch1. Inactivation of Notch1 further inhibits the activation of NLRP3 inflammasome and M1 macrophage polarization, and reduces MMP activity, thus slowing the genesis and development of AAA. This study also uncovered a new inflammatory mechanism of endogenous IMD inhibiting the occurrence of AAA, which provides new directions for therapeutic targets of AAA.

## MATERIALS AND METHODS

### Materials

Synthetic human IMD_1–53_, rat IMD_17–47_ and AngII were from Phoenix Pharmaceuticals (Belmont, CA, USA). Alzet Mini-osmotic pumps (Alzet model 1004) were from DURECT Corp. (Cupertino, CA, USA). DAPT, LPS, IFN-γ, IL-4, PI3K/Akt inhibitor LY294002, cAMP/PKA inhibitor H89 and cGMP/PKG inhibitor Ly83583 were from Sigma (MO, USA). Primary antibodies for Notch1 (ab52627), hes1 (ab71559), NICD (ab8925), iNOS (ab3523), NLRP3 (ab214185), caspase-1 (ab1872), CD68 (ab955), CD16 (ab203883), Arg1 (ab91279), CD206 (ab64693, ADAM10 (ab1997), ADAM17 (ab2051), IL-1β (ab2105), IL-10 (ab9969) and IL-18 (ab191860) were from Abcam (Cambridge, UK). Primary antibodies for p-Akt (Ser473) (4060) and Akt (9272) were from Cell Signaling Technology(CST, USA). Primary antibodies for β-actin (sc-1616), GAPDH (sc-25778), ASC (sc-514414), and all secondary antibodies were from Santa Cruz Biotechnology (Santa Cruz, CA, USA). Other chemicals and reagents were of analytical grade. Mouse macrophage line Raw264.7 were bought from American Type Culture Collection (ATCC, USA).

### IMD transgenic and knockout mice

IMD transgenic and knockout mice were generated by Model Animal Research Center of Nanjing University. All animals were maintained at C57BL/6 genetic background, kept under specific pathogen-free conditions, approved by the Animal Care and Use Committee of Peking University Health Science Center. Male mice 8-12 weeks old were used for experiments.

### AngII-induced AAA model

ApoE^-/-^ mice in a C57BL/6 background were from the Animal Center, Peking University Health Science Center (Beijing). All animal care and experimental protocols complied with the Guide for the Care and Use of Laboratory Animals published by the US National Institutes of Health (NIH Publication, 8^th^ Edition, 2011). ApoE^-/-^ mice (5 months old, male) were randomly assigned to 4 groups for treatment: infusion of saline; AngII (1000 ng/kg/min) [7]; AngII plus IMD_1-53_ (300 ng/kg/h) [28]; and AngII plus DAPT. AngII, saline and IMD_1-53_ were infused for 28 days. DAPT was administered 3 days before AngII infusion. AngII and IMD_1-53_ were administered subcutaneously via an Alzet Mini-osmotic pump. Four weeks later, surviving mice were intraperitoneally injected with pentobarbital (50 mg/kg) for anesthetization.

### CaCl_2_-induced AAA model

Briefly, as described previously [48], C57BL/6 and IMD transgenic and knockout mice were anesthetized with an intraperitoneal injection of pentobarbital (40 mg/kg) before undergoing laparotomy. The abdominal aorta between the renal arteries and bifurcation of the iliac arteries was isolated from the surrounding retroperitoneal structures. The AAA model was induced with extraluminal CaCl_2_. After baseline measurements, 0.5 mol/L CaCl_2_ was applied to the external surface of the aorta for 15 min. The aorta was rinsed with 0.9% sterile saline and the incision was closed. NaCl (0.9%) was substituted for CaCl_2_ in sham control mice.

### Cell culture

BM macrophages were extracted and cultured as previously described [7] with minor modification. Mouse tibias were collected and the BM was flushed with Dulbecco’s modified Eagle’s medium (DMEM; Gibco, USA) and differentiated into primary BMDMs. Cells were cultured in DMEM containing 10% fetal bovine serum (FBS; Hyclone, USA) and incubated at 37° C in a humidified atmosphere containing 5% CO_2_. Macrophages were stimulated with IFN-γ and IL-4 to induce M1 and M2 macrophage polarization, respectively [30].

The mouse peritoneal macrophages were collected and cultured as previously described [49] with minor modification. The mouse were injected intraperitoneally with 2 ml of brewer modified thioglycollate medium (BD, USA). Three days later, cells were harvested by peritoneal lavage with 5 ml RPMI-1640 medium (Gibco, USA). Then cells were washed with cold RPMI-1640 medium and seeded on plastic plates. After two hours incubation, the non-adherent cells were washed with RPMI-1640 medium and the adherent cells were monolayer macrophages.

### Wound healing assay

The mouse peritoneal macrophages was used in wound healing assay. Macrophages were cultured in 6-well plates and starved for 12 hours in medium supplemented with 0.5%FBS. Then macrophages were scraped, washed with PBS and incubated under 37° C in the presence of LPS or IMD_1-53_ for 24 hours in an atmosphere containing 5% CO_2_. Images were captured through a Olympus microscope.

### Histology and immunofluorescence of mice

Mice were killed and the abdominal aortas were harvested, fixed for 12 hr and embedded in paraffin, and cross-sections (6 μm) were prepared. Paraffin sections were stained with hematoxylin and eosin (HE) and Verhoeff-van Gieson (VVG).

For immunofluorescence assay, freshly cut OCT-embedded aorta sections were incubated with the antibodies anti-CD68 (1:100 dilution) for 1 hr at 37° C, then at 4° C overnight, washed with PBS, then incubated with secondary TRITC-conjugated goat anti-rabbit IgG (1:300 dilution). IgG was a negative control. DAPI was used to stain the nuclei.

### Immunohistochemistry of human and mice

Human and mouse AAA aortas were used in immunohistochemistry assay. Surgical specimens were obtained from AAA patients undergoing open surgical repair at Beijing An Zhen Hospital. The control aortic samples were obtained from heart transplantation donors (without AAA) at Beijing An Zhen Hospital. Informed consent was obtained from all patients according to protocols approved by the Medical Ethical Committee of Beijing An Zhen Hospital that comply with the principles outlined in the Declaration of Helsinki.

First, abdominal aortas were fixed in paraformaldehyde for 12 hr and embedded in paraffin, and cross-sections (6 μm) were prepared. For immunohistochemistry, paraffin sections from human or mouse aortas were incubated at 37° C for 1 hr, then at 4° C overnight with primary antibodies for hes1 (1:300 dilution), NICD (1:100 dilution), iNOS (1:200 dilution), Arg1 (1:300 dilution) or ASC (1:100 dilution). On day 2, arterial sections were rinsed with 0.01 mol/L phosphate buffered saline (PBS), then incubated with horseradish peroxidase-conjugated secondary antibodies diluted in 0.1 mol/L PBS for 1 hr at room temperature and redyeing the nucleus with hematoxylin. Normal serum was a negative control.

### In situ zymography

Freshly OCT-embedded mouse aorta sections (7.00 um) were used to detect MMP activity. Sections were incubated in a dark and humid chamber at 37° C for 24 hr with MMP fluorogenic substrate DQ–gelatin–FITC (Invitrogen), then washed with PBS. Negative controls were incubated with PBS.

### Western blot analysis

Aortic tissue or cells were homogenized in lysis buffer. Then equal amounts of total protein were resolved by 10% or 15% SDS-PAGE (15% for small proteins). Proteins were transferred to a nitrocellulose membrane and blocked with 5% nonfat dried milk for 1 hr, then incubated with the primary antibodies β-actin (1:4000 dilution), GAPDH (1:2000), NLRP3 (1:1000), caspase-1 (1:500), ASC (1:1000), Arg1 (1:1000), IL-1β (1:1000), IL-18 (1:1000), IL-10 (1:200), CD16 (1:300), CD206 (1:1000), Notch1 (1:500), hes1 (1:1000), NICD (1:500), iNOS (1:500), ADAM10 (1:500), ADAM17 (1:500), p-Akt (1:1000) or Akt (1:1000) overnight at 4° C, then with horseradish peroxidase-conjugated secondary antibody for 1 hr. Protein expression was analyzed by using NIH ImageJ and normalized to β-actin expression. All experiments were repeated at least 3 times.

### Quantitative real-time PCR

Total RNA from aortic tissue and BMDMs was extracted with Trizol (Applygen, Beijing) and reverse transcribed by using a reverse transcription system (Applygen, Beijing). Real-time PCR amplification included Applied Biosystems 7500 fast PCR System (Life Technologies, USA) and SYBR Green I reagent (Tiangen Biotech, Beijing). The cycle threshold (Ct) was determined as the number of PCR cycles required for a given reaction to reach an arbitrary fluorescence value within the linear amplification range. Relative quantification was performed according to the 2^-ΔΔCt^ method, with GAPDH as a reference. The primers for real-time PCR were in Table 1.

**Table 1:** Primer sequence for RT-PCR.

**Target**		**Sequence**
Mouse *Notch1*	Forward	5’-ACCCACTCTGTCTCCCACAC-3’
	Reverse	5’-GCTTCCTTGCTACCACAAGC-3’
Mouse *Dll4*	Forward	5’-TTAAGCACTTCCAGGCAACC-3’
	Reverse	5’-ACCACTGCCGCTATTCTTGT-3’
Mouse *hes1*	Forward	5’-GGCGAAGGGCAAGAATAAAT-3’
	Reverse	5’-TGCTTCACAGTCATTTCCAGA-3’
Mouse *hey1*	Forward	5’-GGAGGGTCAGCAAAGCATTA-3’
	Reverse	5’-CTCCCTTCACCTCACTGCTC-3’
Mouse *Adam10*	Forward	5’-CCAGCTCTGATGGCAAAGAT-3’
	Reverse	5’-CACTGAACTGCTTGCTCCAC-3’
Mouse *Adam17*	Forward	5’-TGGCAAATGTGAGAAACGAG-3’
	Reverse	5’-AAACCAGAACAGACCCAACG-3’
Mouse *Il6*	Forward	5’-GCCTTCTTGGGACTGATGCT-3’
	Reverse	5’-TGCCATTGCACAACTCTTTTC-3’
Mouse *Mcp1*	Forward	5’-AGGGACTGAGGCACTCCAGA-3’
	Reverse	5’-TGACGACGAGACTTCCAGACTACA-3’
Mouse *Ifng*	Forward	5’-TGAGACAATGAACGCTACACACT-3’
	Reverse	5’-GTCACCATCCTTTTACCAGT-3’
Mouse *Il12*	Forward	5’-CTGTGCCTTGGTAGCATCTATG-3’
	Reverse	5’-GCAGAGTCTCGCCATTATGATTC-3’
Mouse *Il10*	Forward	5’-GCTCTTACTGACTGGCATGAG-3’
	Reverse	5’-CGCAGCTCTAGGAGCATGTG-3’
Mouse *iNOS*	Forward	5’-GGAGTGACGGCAAACATGACT-3’
	Reverse	5’-TCGATGCACAACTGGGTGAAC-3’
Mouse *Arg1*	Forward	5’-TTTTTCCAGCAGACCAGCTT-3’
	Reverse	5’-AGAGATTATCGGAGCGCCTT-3’
Mouse *Il1b*	Forward	5’-GCAACTGTTCCTGAACTCAACT-3
	Reverse	5’-ATCTTTTGGGGTCCGTCAACT-3
Mouse *Tnf*	Forward	5’-CCCTCACACTCAGATCATCTTCT-3’
	Reverse	5’-GCTACGACGTGGGCTACAG-3’
Mouse *Gapdh*	Forward	5’-ACTTTGTCAAGCTCATTTCC-3’
	Reverse	5’-TGCAGCGAACTTTATTGATG-3’

### Statistical analysis

GraphPad Prism v5.00 for Windows (GraphPad Software Inc, San Diego, CA, USA) was used for analysis; data are expressed as mean ± SD. First, Kolmogorov-Smirnov test was used to evaluate the normality of data distribution (*P*>0.1) and F-test was used to compare variances (*P*>0.1 take for equal variance). Then Student t test was used to compare 2 groups. One-way ANOVA was used to compare more than 2 groups, and when significant (*P*<0.05), the Tukey HSD test was used to test for differences between groups. *P*<0.05 was considered statistically significant. Fisher’s exact test was used for the incidence of aneurysm. *P*<0.05 was considered statistically significant.

## Supplementary Material

Supplementary Figures
