# Supplementary material for: Inhibition of Notch1-mediated inflammation by intermedin protects against abdominal aortic aneurysm via PI3K/Akt signaling pathway
Source: Aging (Albany NY). 2021 Feb 1;13(4):5164–84. doi: 10.18632/aging.202436 (PMC7950288; doi:10.18632/aging.202436)
Supplement: Supplementary Figures [file aging-13-202436-s001.pdf]

SUPPLEMENTARY FIGURES

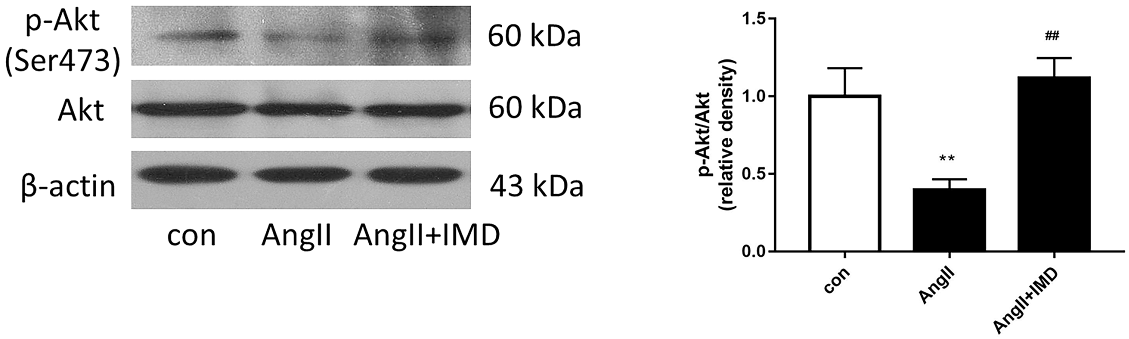

Supplementary Figure 1. IMD reversed the levels of Akt phosphorylation in AngII-induced Raw264.7 macrophages. Western blot analysis of Akt phosphorylation (p-Akt) in AngII-treated Raw264.7 macrophage. n=3, Data are mean ± SD. **P<0.01 vs. Control. ##P<0.01 vs. AngII.

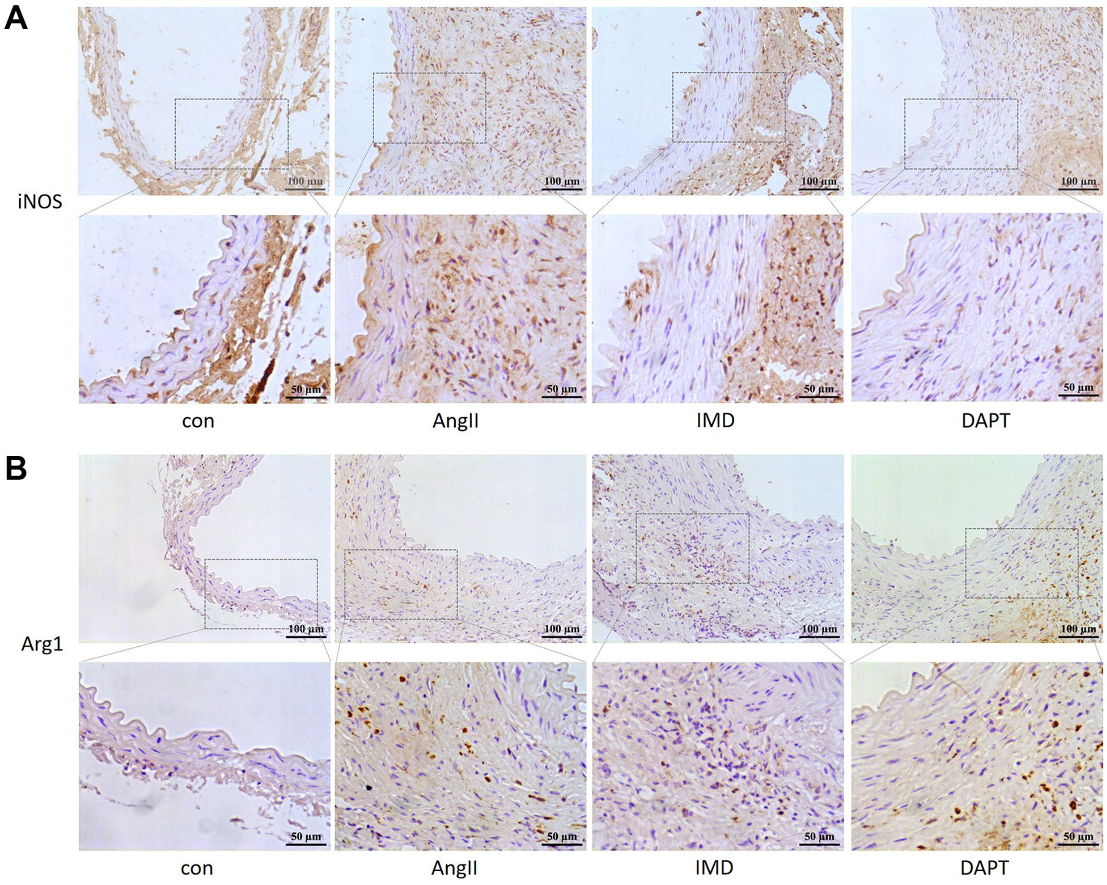

Supplementary Figure 2. IMD inhibited macrophage polarization *in vivo*. (**A**, **B**) Immunohistochemistry of the protein expression of iNOS and Arg1 in aortas of mice. Scale bar, 100 μm, 50 μm. Boxes and arrows show enlarged areas.
